# Efficacy of Technology-Based Cognitive Rehabilitation Tools for Cancer-Related Cognitive Impairment in Non-CNS Cancer Patients: A Systematic Review

**DOI:** 10.3390/healthcare14020239

**Published:** 2026-01-18

**Authors:** Benedetta Capetti, Serena Sdinami, Jenny Luisi, Lorenzo Conti, Roberto Grasso, Gabriella Pravettoni

**Affiliations:** 1 Applied Research Division for Cognitive and Psychological Science, European Institute of Oncology IRCCS, 20141 Milan, Italy; serena.sdinami@ieo.it (S.S.); jenny.luisi@ieo.it (J.L.); lorenzo.conti@ieo.it (L.C.); roberto.grasso@ieo.it (R.G.); gabriella.pravettoni@ieo.it (G.P.); 2 Department of Oncology and Haemato-Oncology, University of Milan, 20122 Milan, Italy

**Keywords:** cancer-related cognitive impairment, cognitive rehabilitation, computerized cognitive rehabilitation, digital cognitive tools, efficacy, non-CNS cancer

## Abstract

**Background**: Cancer-related cognitive impairment (CRCI) is a significant concern for individuals with non-central nervous system (non-CNS) cancers, affecting memory, attention, executive functions, and processing speed. Non-pharmacological interventions, including digital cognitive rehabilitation, have shown promise in addressing CRCI. This systematic review investigates the efficacy of digital and computerized cognitive rehabilitation interventions in improving cognitive outcomes in non-CNS cancer patients. **Method**: A systematic search of the EMBASE, Scopus, and PubMed databases was conducted to identify studies on digital and computerized cognitive rehabilitation for non-CNS cancer patients. Studies were included if they involved computerized and digital cognitive rehabilitation for oncological patients and assessed the efficacy of the intervention. A total of 11 studies were selected, including randomized controlled trials and quasi-experimental designs. The quality of the studies was assessed using the Mixed Methods Appraisal Tool (MMAT). Data was synthesized using a narrative descriptive approach, and the results were summarized in a descriptive table. **Results**: The most frequently assessed cognitive domains included working memory, attention, executive functions, and processing speed. The majority of studies (*n* = 11) demonstrated significant immediate improvements in cognitive functions, particularly in working memory, executive functions, attention, and processing speed. Short-term follow-up (1–5 months) showed partial maintenance of these improvements, while long-term effects (6 months to 1 year) were more variable. Improvements in episodic memory were less consistent, particularly among breast cancer survivors. **Discussion**: Digital and computerized cognitive rehabilitation appears to be an effective intervention for CRCI, providing immediate cognitive benefits and some lasting improvements, especially in domains such as memory and attention. However, long-term effects remain variable, and further research is needed to explore the optimal duration of interventions and the potential advantages of personalized rehabilitation approaches.

## 1. Introduction

Cancer-related cognitive impairment (CRCI) refers to a spectrum of cognitive difficulties experienced by individuals with non-central nervous system (non-CNS) cancers [1]. CRCI primarily affects short-term and long-term memory, attention, executive functions, and processing speed, and is also associated with both structural and functional brain alterations [2,3,4].

The prevalence of CRCI among cancer patients is substantial. Recent evidence suggests that between 17% and 75% of breast cancer patients experience cognitive difficulties following oncological treatments [5]. These impairments may in fact emerge both during or after treatment and are influenced by multiple factors, including treatment type (chemotherapy, hormonal therapy, immunotherapy, and targeted therapy), level of inflammation throughout the body, and psychological distress experienced by the individual [6]. The impact of CRCI on patients’ daily life is significant, often resulting in difficulties in occupational performance, social functioning, and reduced self-esteem [7,8].

To address these challenges, several non-pharmacological interventions have been implemented, such as psychological support, cognitive rehabilitation, physical activity, and lifestyle adjustments, all of which showed effectiveness in mitigating CRCI-related symptoms [9]. Among these approaches, cognitive rehabilitation represents a non-pharmacological therapeutic intervention aimed at restoring cognitive functions and compensating for cognitive deficits [10]. It typically involves targeted exercises designed to retrain or strengthen specific cognitive domains, alongside the acquisition of compensatory strategies to manage persistent impairments [11]. Cognitive rehabilitation is inherently individualized and seeks to improve memory, attention, perception, problem-solving, and other high-order cognitive functions [11].

In recent years, digital technologies—such as mobile applications, internet platforms, wearable devices, virtual reality, and artificial intelligence (AI)—have emerged as promising forms of non-pharmacological tools for the management of cognitive impairment among cancer patients [9]. In particular, AI-driven technologies offer advantages such as individualized adaptation, flexibility, interactivity, continuous data tracking, monitoring, and feedback, thus enhancing the delivery of traditional cognitive interventions [12]. Moreover, evidence from diverse clinical populations suggests that digital cognitive training improves accessibility, continuity of care, and patient comfort while achieving cognitive outcomes comparable to, or in some cases superior to, those obtained through conventional interventions [13]. In this context, the comprehensive review conducted by Shen and collaborators (2025) [14] highlighted the effectiveness of digital cognitive rehabilitation in neurological populations, demonstrating improvements in executive functioning, memory, attention, and overall quality of life. Consistent findings were also reported in a 2025 meta-analysis that quantitatively evaluated digital cognitive interventions in patients with traumatic brain injury: the results showed that digital interventions significantly improved global cognitive function, executive function, attention, and social cognition [15]. The study found that these digital interventions significantly improved global cognitive function, executive function, attention, and social cognition [16]. Collectively, these findings suggest that digital cognitive rehabilitation enhances cognitive outcomes, paving the way for more accessible and personalized interventions across diverse patient populations.

Building on the demonstrated effectiveness of digital and computerized cognitive interventions in other clinical populations, the present review aims to describe and synthesize the current evidence on the efficacy of technology-based cognitive rehabilitation interventions for CRCI in non-CNS cancer patients.

## 2. Methodology

### 2.1. Clarity and Accessibility

This systematic review has been conducted following the Preferred Reporting Items for Systematic Reviews and Meta-Analyses (PRISMA) guidelines [1,17]. In addition, a review protocol was developed and registered in the PROSPERO International Prospective Register of Systematic Reviews database in November 2025 (Registration ID: CRD420251233422).

### 2.2. Inclusion and Exclusion Criteria

Studies were included if they met the following criteria: (i) at least one intervention implemented in the study was a computerized cognitive rehabilitation approach; (ii) the study population consisted of cancer patients with non-CNS tumors; (iii) the study evaluated the effectiveness of the intervention; (iv) the study included at least one objective cognitive assessment to measure cognitive outcomes.

Studies were excluded if they met any of the following criteria: (i) the study assessed only feasibility, acceptability, or adherence to the intervention without measuring its effectiveness; (ii) the study provided only qualitative measures of the rehabilitation intervention; (iii) the study included only psycho-educational interventions, even if delivered via web/computer; (iv) the study included patients with CNS tumors. However, patients with brain metastases were included if the primary tumor was non-CNS.

### 2.3. Information Source and Search Strategy

In the present study, three databases were consulted for eligible studies: EMBASE, Scopus, and PubMed. An online literature search was performed on 23 June 2025. The research question was formulated on the basis of terms related to the following Population, Intervention, Comparison, Outcome, Study (PICOS) criteria [18]:

1. Population: Oncological patients;
2. Intervention: Available computerized tools used for neuro-cognitive rehabilitation, which targeted one or more specific cognitive domains;
3. Comparison: Control groups subjected to no intervention OR subjected to non-computerized rehabilitation interventions;
4. Outcome: Efficacy of computerized interventions, which yielded both self-perceived and objectively measured assessments of cognitive rehabilitation;
5. Study: Quantitative and mixed-methods designs.

Records were, therefore, searched for using MeSH terms closely related to the PICOS criteria, such as “Cognitive Impairment”, “Chemotherapy-Related Cognitive Impairment”, “Cancer Survivors”, “Neoplasms”, “Rehabilitation”, and “Cognitive Training”.

Boolean operators “AND” and “OR” were used to combine keywords. Simultaneously, we limited the search to non-CNS tumors, to only studies written in the English language, and to the adult oncological population. Moreover, the search was conducted without any restrictions on publication year.

The query was developed in consultation with a librarian at the European Institute of Oncology; it was first designed in PubMed, and then the appropriate MESH/thesaurus terms and formats were translated and adapted for the other two databases. The three final search strategies used are reported in Supplementary Material Table S1.

### 2.4. Study Selection Process

All the studies obtained through the search strategy were imported into the Rayyan software (version of 2023) to streamline the processes of screening, analysis, and selection. Within Rayyan, duplicate records were automatically detected by the system and then manually verified and removed.

Four reviewers (BC, SS, JL, and LC) independently evaluated the eligibility of the collected studies. In the first phase, titles and abstracts were screened and labeled as “included,” “excluded,” or “uncertain.” This preliminary assessment was performed on Rayyan using the blind-on setting, ensuring that the reviewers’ judgments remained independent.

To strengthen methodological rigor and enhance reliability, each reviewer’s assessments were subsequently cross-checked by another member of the review team. After disabling the blind mode, any disagreements were resolved through discussion among the reviewers or, when consensus could not be reached, by consulting a fifth reviewer (RG).

Finally, all the studies categorized as “included” or “uncertain” underwent full-text analysis for a more in-depth evaluation. In this final screening phase, the articles were evenly divided among the four reviewers (BC, SS, JL, and LC). Any uncertainties regarding inclusion or exclusion were discussed collectively until a final decision was made. A last check on the included studies was made by a fifth reviewer (RG).

### 2.5. Data Extraction

Four reviewers (BC, SS, JL, and LC) carried out the extraction of relevant data from all the studies that met the inclusion criteria. Information was collected regarding the study’s author and publication year, country of origin, methodological design, aims, characteristics of the population and sample size, participants’ mean age, cancer type, procedural aspects, cognitive domains investigated, type of cognitive rehabilitation, and the main results. Extracted data were then organized in a Microsoft Excel spreadsheet (version 2016) to facilitate comparison, data synthesis, and data analysis. To ensure data reliability, a fifth reviewer (RG) performed an additional verification of the extracted material.

Throughout this stage, frequent discussions were held among the reviewers to confirm the coherence, accuracy, and completeness of the extracted information. Given the heterogeneity of study designs, interventions, outcome measures, and follow-up durations, a quantitative meta-analysis was not feasible. Therefore, a narrative descriptive synthesis was conducted. Data were summarized using descriptive tables and systematically compared across studies in terms of intervention characteristics, cognitive domains assessed, and reported outcomes.

### 2.6. Quality Assessment Results

The methodological quality of the studies included in this review was appraised using the Mixed Methods Appraisal Tool (MMAT) [19], with independent ratings provided by four authors (BC, SS, JL, and LC).

The MMAT evaluates five types of studies: qualitative, quantitative, randomized controlled trials, non-randomized quantitative studies, quantitative descriptive studies, and mixed-methods studies. The evaluation begins with two general screening questions, applicable to all study types regardless of methodology: “Are the research questions clearly defined?” and “Does the data collected allow for addressing the research questions?”. These two screening questions, which apply to all study designs, are followed by five additional questions that vary according to the specific study design. For quantitative randomized controlled trials and non-randomized controlled trials, five criteria are assessed. Responses to the questions are categorized as “yes,” “no,” and “can’t tell.” The “can’t tell” option applies when the article does not provide enough information to answer with a definitive “yes” or “no”. Quality was categorized as low (MMAT score, 2–4), moderate (MMAT score, 5–6), or high (MMAT score, 7).

## 3. Results

### 3.1. Study Selection and Characteristics

The database search conducted across PubMed, EMBASE, and Scopus yielded a total of 3313 records. Following the removal of 223 duplicate entries, 3090 studies remained for the initial screening, which was based on titles, abstracts, and keywords. Of these, 3032 records were excluded as they did not meet the inclusion criteria.

A subsequent full-text assessment was performed on the remaining 58 articles, after which 46 studies were excluded due to non-compliance with the predefined eligibility criteria. More specifically, of the 46 studies: 19 studies were excluded due to wrong study design, as they were conference abstracts or study protocols; 1 study was excluded as it was not written in English language; 1 study was excluded because it employed eye-tracking as cognitive assessment tool rather than standardized neuropsychological tests; 9 studies were excluded as they did not include a digital cognitive rehabilitation in their intervention; and 17 studies were excluded as they investigated the acceptability or feasibility of the intervention rather than the efficacy of the intervention itself. Therefore, ultimately, a total of 11 studies were retained for inclusion in the review.

**Figure 1:**
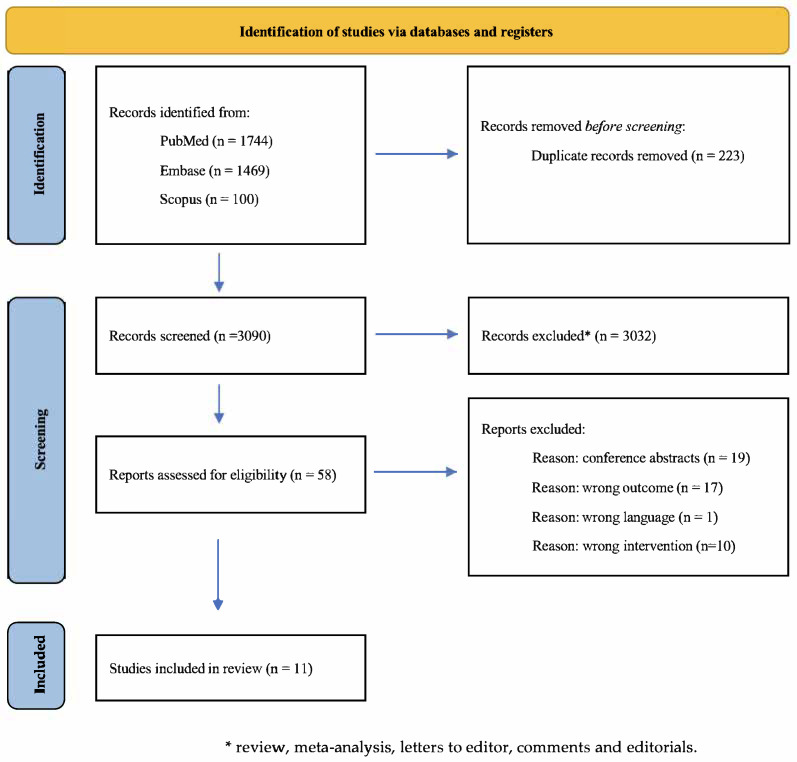
PRISMA (Preferred Reporting Items for Systematic Reviews and Meta-Analyses) flow chart of the study selection process.

The screening and selection process was primarily conducted by three reviewers (BC, SS, and JL) and independently verified by two additional reviewers (LC and RG). A detailed overview of the study selection process, together with specific exclusion reasons, is presented in Figure 1.

#### 3.1.1. Quality Assessment of the Included Studies

Three studies achieved a high-quality assessment (*n* = 3) while the remaining nine were rated as having moderate methodological quality (*n* = 9). Detailed results from MMAT are reported in the Supplementary Material Table S2.

#### 3.1.2. Sociodemographic Characteristics

The studies included in the current review were published between 2011 and 2025.

Most of the studies (*n* = 4) were conducted in the US, followed by 2 studies conducted in Germany. The final five studies were conducted, respectively, in France, the UK, Spain, Australia, and Israel.

The sample size of the included studies ranged from 13 to 242 participants, with a total of 814 females and 42 males.

The age of participants ranged from 23 to 78 years old, with the average age of participants being approximately 52.5 years. The education levels of participants varied from primary school to university, with the average years of education being approximately 14.7.

The majority of the included studies (*n* = 6) only focused on breast cancer populations, whereas the other 7 studies did not limit the intervention to breast cancer patients cohort, but also included other oncological diagnosis, such as ovarian cancer, Hodgkin’s lymphoma, non-small-cell lung cancer with brain metastases, supraglottic/laryngeal cancer, throat/tongue cancer, anaplastic oligodendroglioma, colon cancer, prostate cancer, lymphoma, multiple myeloma, endometrial cancer survivors, colorectal cancer, and lymphoma. The majority of the included studies (*n* = 7) excluded patients with metastases, while some studies (*n* = 3) did not provide any information on this aspect. Only two studies included patients with metastases: in one study [3], 15 patients (9% of patients included) had non-brain metastases, and in another study [20], only one patient with non-small-cell lung cancer had brain metastases. Further information on the study characteristics can be found in Table 1.

#### 3.1.3. Included Studies’ Design

Seven of the included studies are randomized controlled trials [3,21,22,23,24,25,26], while four studies have a quasi-experimental, pre–post design [20,27,28,29].

**Table 1:** Sociodemographic characteristics and cognitive domains investigated.

First Author, Year	Study Design	Country	Aim	Population (Patients, CG)	Gender (Male, Female)	Age	Education	Cancer Type
[20]	quasi-experimental, pre–post design	USA	to explore the efficacy and feasibility of a combined aerobic and cognitive training intervention on cognitive function in participants undergoing treatment for cancer	AER + COG group (*n* = 9); AER group (*n* = 7); COG group *n* = 5; CON group *n* = 7	6 males; 22 females	(mean, SD) 57.9 ± 8.0	Missing Data	Breast cancer (*n* = 14) Ovarian/breast (*n* = 1) Hodgkin’s lymphoma (*n* = 1) Non-small-cell lung metastasized to the brain (*n* = 1) Supraglottic/laryngeal (*n* = 1) Ovarian (*n* = 2) Throat/tongue (*n* = 1) Anaplastic oligodendroglioma (*n* = 1) Colon (*n* = 1) Breast/colon (*n* = 1) Prostate (*n* = 1) Lymphoma (*n* = 1) Lung (*n* = 1) Multiple myeloma (*n* = 1)
[21]	randomized controlled trial	Germany	to implement a cognitive therapy approach in the rehabilitation of breast cancer patients following adjuvant CHT and to evaluate their effects	*n* = 96; [NPT group *n* = 33 PC group *n* = 34 CG *n* = 29]	96 females	(mean, SD) 49.19 ± 7.71	Apprenticeship: 54 Polytechnic: 19 University: 16 Other: 4 None: 3	Breast cancer (*n* = 96)
[28]	quasi-experimental pre–post design	Spain	to evaluate the safety, feasibility, and preliminary effectiveness of a computerized home-based cognitive stimulation program for breast cancer survivors experiencing CRCI after chemotherapy.	*n* = 13	13 females	Mean 51 years, range (35–67)	40%: college education diploma 20%: high school 40%: general education development or similar	Breast cancer (*n* = 13)
[27]	quasi-experimental; pre–post design	USA	to evaluate the feasibility and acceptability of Neuroflex in cancer survivors with CRCI	*n* = 21	21 females	(mean, SD) 56.19 ± 10.55	Missing Data	Breast cancer survivors (*n* = 17) Ovarian cancer (*n* = 3) Endometrial cancer survivor (*n* = 1)
[22]	randomized controlled trial	USA	to compare training in memory and speed of processing to wait-list control among long-term breast cancer survivors	*n* = 82 [memory training *n* = 26 speed of processing training *n* = 27 control group *n* = 29]	82 females	(mean, SD) 56.5 ± 8.5	Missing Data	Breast cancer (*n* = 82)
[23]	randomized controlled trial	USA	to investigate the feasibility and preliminary effectiveness of a novel, online EF training program in long-term BC survivors.	*n* = 41; [Active group *n* = 21 Wait list *n* = 20]	41 females	Active group: (mean, SD) 55 ± 7 Wait list: (mean, SD) 56 ± 6	Active group: 16 ± 2 years of education; Wait-list: 16 ± 3 years of education	Breast cancer (*n* = 41)
[24]	randomized controlled trial	Germany	to investigate a web-based cognitive training for the immediate post-treatment phase	*n* = 31; [Training group *n* = 16 Control group: *n* = 15]	31 females	Control group: (mean, SD) 54.4 ± 13.8 Training group: (mean, SD) 53.6 ± 11.2	Control group:15.3 ± 2.3 years of education; Training group: 14.5 ± 2.1; years of education	Breast cancer (*n* = 31)
[25]	randomized controlled trial	Israel	To examine the preliminary efficacy of CRAFT combining remote CCT and occupation-based treatment in adults with CRCI.	74 individuals with CRCI [CRAFT *n* = 25 CCT group *n* = 25 TAU group *n* = 24]	18 males; 56 females	CRAFT group: (mean, SD) 48.64 ± 10.26 CCT group: (mean, SD) 51.24 ± 11.70 TAU group: (mean, SD) 54.33 ± 9.50	CRAFT: 15.60 ± 2.14 years of education; CCT: 14.96 ± 2.11 years of education; TAU: 14.17 ± 2.29 years of education	Breast cancer (*n* = 40) Colorectal cancer (*n* = 8) Lymphoma cancer (*n* = 8) Other (*n* = 18)
[26]	longitudinal, randomized controlled trial	Australia	To evaluate a cognitive rehabilitation program (Insight) and compared it with standard care in cancer survivors self-reporting cognitive symptoms.	*n* = 242 [CRP group N = 121; CG *n* = 121]	12 males; 230 females	CRP group: median 52, range (23–74) CG: median 54, range (31–74)	CRP group: median 14, range (8–19) CG: median 12, range (3–19)	Any type of solid cancers (no CNS cancers)
[29]	Longitudinal, quasi-experimental, pre–post design	UK	To investigate the transfer effects of online adaptive cognitive training (dual n-back training) on subjective and objective cognitive markers in a longitudinal design.	*n* = 62 [Intervention group *n* = 31; CG *n* = 31]	62 females	Intervention group: median 49.19, range (34–60) CG: median 47.45, range (36–61)	Intervention group: *n* = 9: Secondary/further education *n* = 18: Higher education CG: *n* = 8: Secondary/further education *n* = 20: Higher education Seven women did not disclose their highest level of education	Breast cancer *n* = 61
[3]	longitudinal, randomized controlled trial	France	to evaluate the impact of computer-assisted CR on cognition, QoL, anxiety, and depression among cancer patients treated with chemotherapy.	*n* = 167 [computer-assisted CR group *n* = 55; exercise at home group *n* = 56; phone call group *n* = 56]	7 males; 160 females	group A (experimental group, computer-assisted CR) = median 51.7, range (35–72) group B (exercise at home) = median 50.9, range (28–78) group C (phone call) = median 50.7, range (24–77)	Computer-assisted CR group: *n* = 4 Primary school *n* = 8 Middle school *n* = 8 High school *n* = 28 University *n* = 7 Unknown Exercise at home group: 4 = Primary school *n* = 8 Middle school *n* = 8 High school University = 28 (50.9) *n* = 8 Unknown Phone call group: *n* = 2 Primary school = *n* = 10 Middle school = *n* = 9 High school *n* = 31 University *n* = 4 Unknown	Computer-assisted CR group Breast cancer, *n* = 47 Digestive cancer, *n* = 4 Hematologic cancer, *n* = 2 Urologic/Gynecologic cancer, *n* = 1 Other, *n* = 1 Exercise at home group Breast cancer, *n* = 48 Digestive cancer, *n* = 2 Hematologic cancer. *n* = 3 Urologic/Gynecologic cancer, *n* = 3 Other, *n* = 0 Phone call group Breast cancer, *n* = 45 Digestive cancer, *n* = 1 Hematologic cancer, *n* = 2 Urologic/Gynecologic cancer, *n* = 7 Other, *n* = 1

Note: USA: United States of America; AER: aerobic; COG: computer-based cognitive; CON: flexibility; CHT: chemotherapy; NPT: neuropsychological training group; PC: computer-based training; CG: control group; CRCI: cancer-related cognitive impairment; BC: breast cancer; CRAFT: Cognitive Retraining and Functional Treatment; CCT: computerized cognitive training; TAU: Treatment as usual; CRP: cognitive rehabilitation program; SD: standard deviation; CNS: central nervous system; CR: cognitive rehabilitation; QoL: quality of life.

#### 3.1.4. Cognitive Rehabilitation Interventions Characteristics

Across the included studies, all the interventions relied on digital, computer-based, or online cognitive rehabilitation, although the structure, duration, and delivery modalities varied considerably. A first group of studies employed structured digital training programs lasting between 6 and 12 weeks, typically involving 3–4 sessions per week [20,21,22,23,24,25,26,27]. These interventions, implemented through computerized cognitive training software, adaptive programs, or small-group digital sessions, provided between 10 and 48 sessions, each lasting 20 to 60 min. Many of these programs incorporated adaptive task difficulty, remote monitoring, and targeted exercises addressing multiple cognitive domains.

In addition, in the study conducted by Chapman and collaborators (2023) [29], participants underwent 12 remote daily training sessions over 2 weeks.

Another study consisted of short-term intensive digital interventions, characterized by daily or near-daily training over a brief period. Specifically, in the study conducted by Tapia and collaborators (2023) [28], the authors administered a concentrated 2-week cognitive stimulation protocol with structured daily activity cycles and pre–post intervention assessments.

In contrast, one study implemented longer-term digital rehabilitation programs, extending them up to 3 months, with 9 sessions [3].

#### 3.1.5. Cognitive Domains Investigated

Across the included studies, a wide range of cognitive domains was assessed to determine the efficacy of digital cognitive rehabilitation interventions. Working memory emerged as the most frequently evaluated domain, being assessed in the majority of studies [3,20,21,23,24,25,26,27,29]. Evaluations typically encompassed both verbal and visuospatial components, and auditory working memory and dual-task performance, with the implementation of n-back paradigms.

Attention was also extensively investigated, including sustained, selective, and divided attention, and alertness [3,20,21,22,24,25,26,28]. Many computerized interventions incorporated tasks specifically designed to assess attentional control, visual tracking, or attentional shifts, reflecting the functional difficulties commonly reported by cancer survivors. Executive functions, encompassing cognitive flexibility, inhibition, planning, dual-tasking, and goal management, represented another core domain of assessment. These functions were particularly targeted in studies that employed adaptive training software or gamified multi-domain programs that inherently engage executive processing [3,20,23,24,28].

Several studies [20,22,23,25,26] also assessed processing speed. Memory outcomes, including immediate and delayed recall, episodic memory, and verbal learning, were also evaluated in multiple investigations [22,27,28].

Additionally, one study also examined verbal fluency [23], while another [3] also incorporated subjective measures of cognitive functioning.

### 3.2. Results of Intervention

In the included studies, cognitive outcomes were assessed at different time points. For ease of reference, we will report the results of the interventions in relation to the time of assessments applied by the included studies. More specifically, we will present the results according to the efficacy of cognitive rehabilitation evidenced at three key time points: immediately after digital cognitive rehabilitation, at short-term follow-up (one to five months after the end of rehabilitation), and at long-term follow-up (six months to one year after the end of rehabilitation).

All the studies assessed cognitive functions at baseline and at the end of the rehabilitation program, providing the results for the immediate effects of rehabilitation on cognitive functions.

Two studies [22,25] also assessed short-term effects of cognitive rehabilitation, respectively, at 2 and 3 months after the end of intervention. Two other studies [21,26] also assessed long-term effects at 6 months after the end of the rehabilitation program, while only one study [29] assessed long-term effects at both 6 months and 1 year after the end of the intervention. The results are summarized in Table 2.

#### 3.2.1. Immediate Effect (Right After Digital Cognitive Rehabilitation)

All of the included studies evaluated the immediate effects of digital cognitive rehabilitation, with the majority reporting acute improvements across several cognitive domains immediately after the rehabilitation program stopped. Specifically, in the study conducted by Vega and collaborators (2023) [27], the use of the Neuroflex platform led to significant improvements in multiple areas of cognitive functioning, including verbal learning, memory, and auditory working memory. Additionally, participants from the same study also reported statistically significant improvements on all four subscales of the Functional Assessment of Cancer Therapy—Cognitive Function (FACT-Cog).

The study by Maeir and colleagues (2023) [25] examined the effectiveness of Cognitive Retraining and Functional Treatment (CRAFT) by combining remote computerized cognitive training (CCT) with occupation-based therapy using BrainHQ. The results revealed significant improvements in perceived cognition and in cognitive performance on speed-of-processing tests immediately after the digital cognitive rehabilitation intervention.

In the study by Dos Santos and others (2020) [3], Group A (computer-assisted neuropsychological rehabilitation) showed the highest proportion of patients with a 7-point improvement in the perceived cognitive impairment (PCI) score at the first time of assessment, followed by Group B (home cognitive exercises) and Group C (telephone follow-up), although the difference was not statistically significant. However, compared to Groups B and C, Group A showed a significantly greater mean difference in the PCI score right after the digital cognitive rehabilitation program, with increased perceived cognitive abilities and significant improvements in working memory.

The CogniFit rehabilitation platform [28] found that participants experienced significant improvements in various cognitive domains, such as executive functions, memory, attention, and concentration levels, right after the computerized cognitive rehabilitation.

The dual n-back training paradigm [29] showed that the intervention led to significant improvements in working memory from day 1 to day 12, right after the digital cognitive rehabilitation program.

**Table 2:** Time of assessment, intervention characteristics, and main results.

First Author, Year	Time of Assessment	Cognitive Domains Investigated	Psychological Domains Investigated	Intervention Characteristics	Main Results
[20]	baseline; post-intervention	general cognitive functioning; processing speed; working memory; executive function; attention; verbal learning and memory; verbal fluidity; perceptual reasoning	Not applicable	AER group: aerobic training + flexibility training; COG group: computerized cognitive training + flexibility training; AER + COG group: combination of aerobic and computerized cognitive training + flexibility training; CON: flexibility training alone. The intervention lasted 12 weeks.	CON group showed an improved verbal fluidity in T2; AER group showed pre to post improvements in logical memory scores the COG group showed no improvements in cognitive function. Absence of any beneficial effects observed in the AER + COG group.
[21]	baseline; post-intervention; 6 months after end of intervention	working memory; sustained attention; story recall; alertness; divided attention	Anxiety; depression; quality of life	Control group: standard neuropsychological training; intervention group: individualized, computer-based training. The intervention lasted 3 weeks.	In “Tonic Alertness” (without warning signal), a clear improvement in performance was observed in all 3 groups between T1 and T2 (*p* = 0.000). In “Story” (delayed recall), a significant time effect was observed between T1 and T2, with higher scores indicating more correctly reproduced text components. The three groups’ results at T3 were similar, with 44.4% (N = 40) of the sample still displaying at least one deficient result.
[28]	baseline; post-intervention	executive functions; memory; attention; concentration	Anxiety; depression; quality of life	Computerized, home-based rehabilitation (CogniFit) The intervention lasted 15 days.	Cognitive functions (Mini-MAC and CAB-CF) show differences pre–post evaluation (*p* < 0.005).
[27]	baseline; post-intervention	working memory; cognitive flexibility; verbal memory	Anxiety; depression	Computerized Neuroflex training [Neuroflex personalizes training with adaptive algorithms that increase difficulty on a trial-by-trial basis, based on three parameters: accuracy, learning curve, and norm-based percentile achievement] The intervention lasted 6 weeks.	Analysis of the sensitivity scores on the N-back task showed a significant effect of condition, with worse performance on the more difficult blocks (*p* < 0.001, *p* = 0.82)
[22]	baseline; post-intervention; 2 months after end of intervention	memory; speed of processing; attention	Mood disturbance; anxiety	Computerized training targeting separate cognitive domains. The intervention lasted 6–8 weeks.	Compared to the wait-list control, the memory training group demonstrated better immediate (*p* = 0.036) and delayed memory performance (*p* = 0.013) at the 2-month follow-up. The speed of the processing group demonstrated better processing speed compared to the wait-list control group post-intervention (*p* = 0.040) and at the 2-month follow-up (*p* = 0.016). Speed of processing training also improved immediate memory at both post-intervention time points (*p* = 0.007 and *p* = 0.004) and delayed memory at the 2-month follow-up (*p* = 0.010).
[23]	baseline; post-intervention	Executive functions; cognitive flexibility; working memory; processing speed; verbal fluency	Anxiety; depression	Online, personalized, computerized cognitive rehabilitation. The intervention lasted 12 weeks.	The active group, compared with the wait-list group, demonstrated significant improvement in the WCST score (*p* = 0.008); the letter fluency (*p* = 0.003); the symbol search (*p* = 0.009); and a trending improvement on the HVLT-R (*p* = 0.07). Digit span scores (*p* = 0.57) and Global BRIEF scores (*p* = 0.22) did not significantly improve. Exploratory analyses suggested significant improvements in BRIEF subscales, including planning and organization (*p* = 0.02) and task monitoring (*p* = 0.03).
[24]	baseline; post-intervention;	attention; working memory; executive function	Not applicable	Online, computerized cognitive rehabilitation. The intervention lasted 14 weeks.	Cognitive impairment significantly improved in the training group (56% vs. 25%; *p* = 0.03), but not in the control group (73% vs. 73%; *p* = 1) in the longitudinal analysis (T1 vs. T2). Specifically, the training group showed statistically significant improvement of executive functions (*p* = 0.004). No effects of training on subjective cognitive deficits or PROMs were observed. Comparing cross-sectional cognitive performance at follow-up (T2), the training group showed a significantly lower rate of cognitive impairment overall (*p* = 0.007) and a better cognitive performance for executive function (*p* = 0.04) compared to the control group.
[25]	baseline; post-intervention; 3 months after end of intervention	attention; speed of processing; visual working memory; attentional control	Not applicable	Online, personalized, computerized cognitive rehabilitation [Brain HQ]. The intervention lasted 12 weeks.	A significant time × group interaction was found on the visual SOP task (*p* = 0.009). Post hoc analysis revealed significant differences between baseline and post-intervention scores for the CRAFT (*p* = 0.002) and for the CCT (*p* = 0.000) groups, but not for the TAU group (*p* = 0.392). No significant time × group interaction effects were found for two other cognitive assessments, examining attentional control and auditory SOP. A significant time × group effect was found for the FACTcog scale (*p* = 0.014). Post hoc analysis revealed significant differences (*p* < 0.05) between baseline and post-intervention scores for all groups. However, the mean change in both CRAFT and CCT groups was more than double that of the mean change in the TAU group.
[26]	baseline; post-intervention; 6 months after end of intervention	visual precision; divided attention; working memory; field of view; visual processing speed	Anxiety; depression	Computerized neurocognitive learning program The intervention lasted 15 weeks.	Statistically significant differences in all FACT-COG subscales in the CRP group were observed at T2, when compared with the CG (*p* < 0.001), and less PCI in the CRP group (*p* < 0.001). Perceived cognitive abilities were significantly better in the CRP group (*p* < 0.001), CRP group also reported less impact on their QoL from PCI (*p* = 0.02). At T3, a decrease in PCI was observed in the CRP group (*p* = 0.001), as perceived cognitive abilities were significantly better in the CRP group (*p* < 0.001); CRP group also reported less impact on their QOL from PCI (*p* < 0.001).
[29]	baseline; post-intervention; 6 months after end of intervention; 1 year after end of intervention	working memory capacity; inhibitory control	Anxiety; depression	Computerized, adaptive cognitive training [Standard versions of dual n-back training and dual 1-back training]. The intervention lasted 2 weeks.	Intervention group: The dual n-back training improved working memory from day 1 (M = 1.72, SD = 0.40) to day 12 (M = 2.47, SD = 0.83), with a significant difference (M = 0.75, *p* < 0.001). Active control group: The dual 1-back group showed high accuracy from day 1 (M = 94%) to day 12 (M = 96%). The intervention group showed greater improvement over time (M difference = 7.10) compared to the active control group.
[3]	Subjective and objective cognitive assessments were completed: at baseline (T0); at the end of the CR program (T3). Subjective cognitive assessments were also completed: 1 month (T1) and 2 months (T2) after initiating the CR program.	attention; memory; executive functions	Anxiety; depression	Group A: Computer-assisted CR [RehaCom]. Group B: Home-based cognitive exercises. Group C: Phone follow-up. The intervention lasted 2 months.	Group A had the highest proportion of patients with a 7-point PCI improvement (75%), followed by groups B (59%) and C (57%), but the difference was not statistically significant (*p* = 0.13). Compared with groups B and C, the mean difference in PCI score was significantly higher in group A (*p* = 0.02), with better perceived cognitive abilities (*p* < 0.01) and a significant improvement in working memory (*p* = 0.03). Group A reported higher QoL related to cognition (FACT-Cog QoL) (*p* = 0.01).

Note: AER: aerobic; COG: computer-based cognitive; CON: flexibility; CR: cognitive rehabilitation; PCA: perceived cognitive ability; Mini-MAC: mini-Mental Adjustment to Cancer Scale; CAB-CF: cognitive Assessment for Chemo Fog Research; WCST: Wisconsin card sorting test; BRIEF: Behavioral Rating Inventory of Executive Function; HVLT-R: Hopkins Verbal Learning Test Revised; PROMS: patient-reported outcomes; SOP: speed of processing; CRAFT: Cognitive Retraining and Functional Treatment; CCT: computerized cognitive training; TAU: Treatment as usual; FACTcog: Functional Assessment of Cancer Therapy—Cognition; PCI: perceived cognitive impairment; QoL: quality of life; CRP: cognitive rehabilitation program.

Research by Kesler and collaborators (2013) [23] on online executive function training led to significant improvements in cognitive flexibility, verbal fluency, and processing speed, with marginally significant downstream improvements in verbal memory right after the intervention.

Kleinknecht and others (2024) [24] conducted a study on gamified computerized training, which showed substantial reductions in executive function and attention impairments after the digital cognitive rehabilitation. However, while the training had positive effects on attention and executive functions, it did not lead to significant improvements in the working memory domain.

The web-based Insight program [26] also yielded positive results immediately after the rehabilitation. Indeed, the computerized neurocognitive learning group (CRP) showed statistically significant improvements compared to the control group (CG) in all FACT-Cog subscales. The CRP group also reported significantly less PCI and experienced less impact of PCI on their quality of life. However, there were no significant differences between groups in the total score or in the six specific cognitive domains.

Peterson and colleagues (2018) [20] investigated the combined effect of aerobic and cognitive training and found that the aerobic training alone (AER) group showed significant improvements in logical memory scores from pre- to post-intervention, while the computerized cognitive training alone (COG) group showed no improvements in cognitive function right after the intervention. Moreover, no beneficial effects were observed in a third group that combined AER and COG.

Lastly, Weis and others (2009) [21] found no significant differences between the neuropsychological training (NPT) group and the computer-based training (PC) group right after digital cognitive rehabilitation. Alertness improved in all three groups (NPT, PC, and control group), while delayed story recall improved significantly more in the NPT group compared to the control group after the intervention.

#### 3.2.2. Short-Term Effects (One to Five Months Post Digital Cognitive Rehabilitation)

Two studies measured the short-term effects of cognitive rehabilitation at 2 and 3 months after the intervention ended.

Firstly, Von Ah and colleagues (2012) [22], while comparing cognitive training among long-term breast cancer survivors and wait-list controls, found that the intervention group demonstrated better immediate and delayed memory performance at the 2-month follow-up assessment. The authors also found better processing speed in the intervention group compared with wait-list controls.

Maeir and collaborators (2023) [25], in their longitudinal design, explored the efficacy of CRAFT on perceived cognitive functions, and speed of processing and visual working memory. The original sample size comprised 74 participants; however, only 32 participants took part in the 3-month follow-up assessment due to COVID-19 restrictions. Statistical analysis found the intervention to be successful in improving immediate perceived cognitive functions, speed of processing, and working memory at the post-intervention assessment: these gains remained visible also at the 3-month mark.

#### 3.2.3. Long-Term Effects (Six Months to Five Years Post Digital Cognitive Rehabilitation)

Three studies measured the long-term effects of cognitive rehabilitation at 6 months after the end of the rehabilitation program. Of these, one study also measured long-term effects at 1 year after the intervention ended.

Weis and collaborators (2011) [21] evaluated the effect of a cognitive rehabilitation program for breast cancer patients who had recently undergone post-adjuvant chemotherapy treatment. Improvements in both working memory and attention were maintained at the 6-month follow-up, even though they tended to decline. Nonetheless, patients still subjectively rated the intervention as being helpful. This is in line with what Bray and collaborators (2017) [26] also found: participants reported less perceived cognitive impairment if subjected to the rehabilitation, highlighting better perceived cognitive abilities and reporting less impact of cognitive deficits on their quality of life. Also, Chapman and colleagues (2023) [29], measuring the cognitive effects of rehabilitation at 6-month and 1-year follow-up, found a perceived improvement of cognitive abilities and performance.

## 4. Discussion

This systematic review aimed to investigate the efficacy of digital and computerized cognitive rehabilitation on CRCI in non-CNS patients. Overall, our results indicate that digital cognitive rehabilitation consistently produces immediate cognitive improvements, with partial maintenance of these improvements in the short term and a more variable duration in the long term. Specifically, improvements were most consistently observed immediately after the intervention and at follow-ups of 3 and 6 months, while effects at 12 months were more variable across studies. Nonetheless, participants still reported the intervention as beneficial even 1 year after the end of the rehabilitation program, evidencing its positive impact on perceived cognitive abilities.

The majority of the included studies investigated the working memory domain, reflecting the central role of working memory in CRCI and its susceptibility to improvement following adaptive digital training. This is in line with the literature that highlights the prevalence of memory and learning difficulties amongst CRCI-affected patients, especially amongst breast cancer populations [30,31,32]. Given that six out of the eleven studies included in our review only had breast cancer patients as the cohort, it is reasonable that the majority of the included rehabilitation focused on these domains. This disproportionate focus on breast cancer limits the generalizability of the findings to other cancer populations, in which CRCI may present with different cognitive profiles and underlying mechanisms.

Moreover, our results highlight the immediate improvements of digital rehabilitation on various cognitive domains compared with wait-list or control groups. More specifically, across all the studies reviewed, the most consistently responsive cognitive domains immediately following digital and traditional interventions were working memory, executive functions, processing speed, and attention, while episodic memory showed more variable improvements, suggesting that some cognitive functions may require more intensive interventions to achieve significant improvements. For instance, episodic memory in breast cancer survivors often exhibits delayed or partial recovery, likely due to neurotoxic effects of chemotherapy on the hippocampus, a key brain area for episodic memory consolidation [33]. In line with these findings, previous research has highlighted that memory, attention, and information processing speed are the most extensively assessed cognitive domains in non-CNS cancer patients [34]. Nonetheless, the absence of standardized thresholds for clinically meaningful change across many of the neuropsychological tests used complicates the interpretation of whether these domain-specific improvements translate into tangible benefits for patients.

Furthermore, our results also show a tendency for these improvements to decline with time, as both short-term and long-term effects highlighted weaker effects of the intervention on cognitive abilities. Nonetheless, participants still report higher subjective, perceived cognitive abilities even at the 1-year follow-up. These results provide preliminary evidence on the duration of cognitive gains by domain and suggest that some domains may retain benefits longer than others. This discrepancy between objective cognitive outcomes and subjective perceptions underscores the importance of incorporating patient-reported outcomes and functional measures when evaluating the real-world impact of digital cognitive rehabilitation. It is worth noticing that these results could have been influenced by high numbers of participants dropping out in longitudinal studies. For instance, at least two of the included studies [24,29] state that their recruitment process was influenced by COVID-19 restrictions, which forced them to consider a smaller sample size for follow-up assessments, diminishing their studies’ power. Also, four other studies included in our review experienced variable loss of participants due to drop out [20,21,24,25]. This lower participation adherence could have influenced the quantitative results related to short- and long-term effects of digital cognitive rehabilitation.

Moreover, five of the included studies involved adaptive, individualized rehabilitation [20,22,24,27,29]. More specifically, these studies all included exercises that evaluated the accuracy of the participant and, through algorithms, adjusted the difficulty of the next exercises of the rehabilitation course. This kind of personalized cognitive rehabilitation, delivered through computerized measures, is known across the literature as CCT [35]. CCT has been found to be a promising alternative to non-pharmacological interventions for CRCI [7,36]. Nonetheless, the results from our review do not show any better effect on cognitive performance when personalized rehabilitation is compared with standard, non-personalized rehabilitation. For instance, three studies that implemented a personalized, digital rehabilitation [22,27,29] either compared their personalized intervention to wait-list control groups or had a longitudinal, quasi-experimental, pre–post design, making it impossible to compare the personalized intervention to a non-personalized one. However, the study conducted by Weis and colleagues (2011) [20], in their randomized controlled trial, compared the efficacy of a neuropsychological rehabilitation between a group of patients who underwent digital, personalized rehabilitation and a group of patients who underwent digital, standard rehabilitation, and found no difference between the two groups in terms of efficacy. Also, Maeir and collaborators (2023) [24] implemented a three-arm randomized control trial and found no difference between the intervention group assigned to the personalized rehabilitation and the intervention group assigned to the non-personalized rehabilitation. These findings suggest that personalization alone may not be sufficient to enhance cognitive outcomes, and that other factors such as intervention intensity, duration, and patient characteristics may play a critical role. Further research is needed to address any significant difference between personalized and non-personalized digital cognitive rehabilitation in patients affected by CRCI.

Finally, rehabilitative interventions of our included studies varied considerably in duration and consistency. For instance, interventions could last anywhere from 15 days [28] to 2 months [3], with a total of rehabilitation hours varying from 10 h [21] to 45 h [27]. However, there is no way of establishing whether the duration of the rehabilitation had any effect on cognitive performance. The literature seems to suggest that longer rehabilitation leads to increased and more sustained cognitive improvements, especially across executive functions [37], and improves functional connectivity and delays structural brain declines [38]. However, to our knowledge, there are no studies further investigating whether the length of cognitive rehabilitation in patients with CRCI has any effect on cognitive performance at immediate, short- or long-term assessments. Further research in the neuropsycho-oncological field regarding this aspect is needed.

A final remark needs to be made regarding the methodological rigor of the included studies and their temporal, geographical, and demographic characteristics, which could all have impacted the generalizability of our results. First, given the disproportionately high representation of women with breast cancer in the included studies, our results might not be generalizable to the entire oncological population and to male oncological patients. Furthermore, 6 out of the 11 included studies were published later than 2020, with 5 studies published in the last two years. This temporal trend indicates a growing focus on cognitive deficits in oncological patients, which is expected to increase in future publications. Our contribution rests, therefore, on relatively new findings across the literature. Moreover, even though the geographical distribution of the included studies spans across four continents (Europe, North America, Asia, and Australia), the previous literature on barriers to inclusivity in oncological research highlights the possibility of biases and systematic practices that may limit the inclusivity and generalizability of results to the entire oncological population [39]. Lastly, a certain degree of methodological transparency of the included studies (see Supplementary Materials Table S2), and the heterogeneity of oncological populations and sometimes small sample sizes, might also have contributed to the weakening of the reliability, robustness, and generalizability of our results, which, therefore, need to be interpreted with caution.

Overall, our results suggest that computerized and digital-based interventions for cognitive rehabilitation are a promising non-pharmacological tool to improve both perceived and objectively measured cognitive deficits related to cancer and cancer treatment. The strongest evidence of efficacy was observed in the domains of working memory and attention, particularly among breast cancer patients, with effects most consistently detected immediately post-intervention and maintained at 3- and 6-month follow-up assessments. In contrast, the findings related to episodic memory were more heterogeneous, and evidence supporting the efficacy of digital cognitive rehabilitation in other cancer populations remains limited. Taken together, our results suggest that digital cognitive rehabilitation may provide clinically meaningful benefits. However, further high-quality research is needed to clarify long-term effectiveness, determine optimal training intensity and duration, and evaluate generalizability across different cancer types and treatment trajectories.

### 4.1. Limitations and Strengths of the Current Review

This systematic review is subject to several limitations: Firstly, our inclusion criteria restricted the selection to studies published in English, which may have resulted in the exclusion of potentially relevant research published in other languages.

In addition, due to the substantial heterogeneity across studies in terms of cancer types, intervention characteristics, outcome measures, and follow-up durations, a quantitative synthesis or meta-analysis was not feasible. Consequently, the findings of this review are based on a narrative descriptive synthesis, which limits the ability to estimate pooled effect sizes and to draw firm conclusions regarding the magnitude of intervention effects.

Another limitation of this review is the decision to include only studies that focused on cognitive rehabilitation, excluding those that incorporated psychoeducation as part of the intervention. This could potentially impact the results, as psychoeducation may play a role in enhancing cognitive outcomes. Additionally, the exclusion of older patients, with the focus being solely on adults, could be considered another limitation. Although this exclusion was intended to minimize confounding variables, it may limit the broader applicability of our findings to all cancer patients. Furthermore, the predominance of breast cancer patients in the studies included may reduce the homogeneity of the sample, underscoring the need for more research that includes diverse populations of non-CNS cancer patients.

Another limitation of this review is the inclusion of patients with non-CNS cancers as a relatively heterogeneous group. Indeed, CRCI may arise from different pathophysiological mechanisms depending on cancer type, disease biology, and treatment exposure [40]. For example, cognitive changes observed in hormone-dependent breast cancer may differ substantially from those experienced by patients with hematological malignancies undergoing intensive chemotherapy [41]. The lack of disease-specific analyses limits the ability to characterize distinct cognitive profiles and mechanisms across cancer types.

Finally, although anxiety and depression were assessed in most of the included studies, three out of eleven did not evaluate these conditions, which may have influenced both cognitive outcomes and the subjective perception of cognitive improvement.

The strengths of this review are primarily reflected in the fact that the majority of the included studies were of high and medium-high quality, utilizing validated and standardized tests. Moreover, specific guidelines were adhered to during the conduct of this systematic review. These aspects highlight a rigorous and transparent approach to research, ensuring that the methodology and objectives of the review were clearly defined from the outset.

### 4.2. Clinical Implications

The findings of this review suggest several clinical implications for the treatment and management of cognitive impairments in non-CNS cancer patients. One of the most notable implications is the fact that digital interventions offer a promising solution for patients who may have limited access to in-person rehabilitation services due to geographical barriers, physical limitations, or time constraints. Moreover, given the significant cognitive improvements observed immediately after the rehabilitation program, healthcare providers should consider integrating digital cognitive rehabilitation into routine oncology care. Indeed, addressing these cognitive impairments early in the treatment process could significantly improve patients’ quality of life and overall well-being.

The results of this systematic review also highlight the importance of tailoring cognitive rehabilitation programs to the individual needs of patients. Since cancer survivors may experience different degrees of cognitive impairments, personalized interventions that target specific cognitive deficits would likely be more effective than one-size-fits-all programs. Healthcare providers should work with patients to assess their cognitive challenges and choose the most appropriate rehabilitation program based on individual needs and preferences [42,43]. In addition, the combination of digital tools, professional support, and tailored rehabilitation plans could provide a holistic approach to improving the cognitive health of cancer survivors. Finally, clinical management should adhere to standardized methods and guidelines that are grounded in a substantial body of research on effective rehabilitation protocols, interventions, and therapeutic strategies [44].

### 4.3. Future Directions

In terms of future directions, it would be useful for future studies to explore more personalized rehabilitation programs with longer follow-up periods in order to assess changes in patients’ cognitive profiles over the long term. Specifically, follow-up assessments of 6–12 months could provide valuable information on the sustainability of cognitive improvements. Furthermore, the integration of more rigorous outcome measures, particularly standardized cognitive assessments, could improve comparability between studies and strengthen the robustness of the findings [34]. Future studies should also systematically include measures of clinical significance, such as assessments of functional outcomes related to daily activities, work performance, and social participation, to ensure that improvements in test scores translate into meaningful benefits for patients’ daily quality of life. Studying the role of digital rehabilitation as part of a broader, multifactorial intervention that includes psychoeducation, physical activity, and social support could also provide valuable insights into its holistic benefits for cancer survivors. Importantly, future research should place greater emphasis on the clinical relevance of cognitive changes by integrating objective neuropsychological assessments with patient-reported outcomes and functional measures. This approach would help determine whether statistically significant improvements in test performance translate into meaningful benefits in daily functioning and quality of life. Establishing clinically meaningful thresholds for commonly used neuropsychological instruments would further enhance the interpretation of cognitive outcomes from a patient-centered perspective. Finally, future research should prioritize disease-specific and treatment-specific studies to better characterize CRCI and to develop more targeted and personalized cognitive rehabilitation approaches.

## 5. Conclusions

This systematic review suggests that digital cognitive rehabilitation may represent a promising approach for addressing CRCI in patients with non-CNS cancer, particularly in the post-treatment phase. However, the current evidence is characterized by methodological heterogeneity, limited sample sizes, and variability in outcome measures, which restrict the strength and generalizability of the conclusions.

While several studies reported statistically significant improvements in cognitive performance, the clinical relevance of these changes and their impact on daily functioning and quality of life remain insufficiently established. Moreover, differences in cancer type, treatment exposure, and intervention characteristics further complicate the interpretation of the findings. Therefore, although digital cognitive rehabilitation appears to be a feasible and potentially beneficial intervention, the available evidence does not yet support firm clinical recommendations. Future well-designed, disease-specific studies with standardized outcome measures, longer follow-up periods, and assessments of clinically meaningful and functional outcomes are needed to clarify the role of digital cognitive rehabilitation in the management of CRCI and to define its place within cancer survivorship care.

## Data Availability

No new data were created or analyzed in this study. Data sharing is not applicable to this article.

## Supplementary Materials

The following supporting information can be downloaded at https://www.mdpi.com/article/10.3390/healthcare14020239/s1. Table S1: Queries used to search for eligible studies across three different databases; Table S2: Quality assessment of the included studies [3,20,21,22,23,24,25,26,27,28,29]; Table S3: Prisma 2020 Checklist; Table S4: Prisma 2020 for abstract checklist.
